# Therapy of human T-cell acute lymphoblastic leukaemia with a combination of anti-CD7 and anti-CD38-SAPORIN immunotoxins is significantly better than therapy with each individual immunotoxin

**DOI:** 10.1054/bjoc.2000.1633

**Published:** 2001-02

**Authors:** D J Flavell, D A Boehm, A Noss, S L Warnes, S U Flavell

**Affiliations:** The Simon Flavell Leukaemia Research Unit, Division of Cancer Sciences, University of Southampton Medical School, Southampton General Hospital, Tremona Rd., Southampton, Hampshire, SO16 6YD, UK

**Keywords:** T-cell leukaemia, combination immunotoxin therapy, SCID mouse

## Abstract

Severe combined immunodeficient (SCID) mice injected i.v. with the human T-ALL cell line CCRF CEM (SCID-CEM mice) develop within 50 days life-threatening multi-organ growth of leukaemia cells. The development of leukaemia in SCID-CEM mice treated with three 10 μg i.v. doses of the anti-CD7 immunotoxin (IT) HB2-SAPORIN or the anti-CD38 IT OKT10-SAPORIN was significantly delayed compared with PBS sham-treated animals but 90% of animals treated with either IT eventually developed disseminated leukaemia cell growth. In contrast treatment of SCID-CEM mice with a combination of both ITs led not only to a significantly greater delay in time to leukaemia development but also in the numbers of animals remaining leukaemia free (60%). The native HB2 and OKT10 antibodies (both murine IgG _1_ antibodies) exerted significant, though relatively weak therapeutic effects, probably mediated through an antibody-dependent cellular cytotoxicity (ADCC) mechanism. Moreover, there was no in vivo additivity of therapeutic effect when both antibodies were used in combination. Apparent, however, was that the combination of HB2-SAPORIN IT with OKT10 antibody led to an intermediate therapeutic effect that was significantly greater than that obtained when either was used alone but significantly less than that obtained when the two IT combination was utilized. This was similarly the case for the combination of OKT10-SAPORIN IT with HB2 antibody though the effect was less pronounced in this instance. This result suggests that the therapeutic effect of IT + antibody treatment results from an additivity between antibody-mediated delivery of saporin combined with a SCID mouse NK cell-mediated ADCC attack on the target cell directed through target cell bound antibody Fc engagement with FcγRIII on the NK cell surface. The combination of both ITs however gave the best therapeutic outcome in SCID-CEM mice probably as the result of (i) delivery of greater amounts of saporin to target CEM cells positive for both CD7 and CD38, (ii) delivery of an effective dose of saporin to CEM cells downregulated or negative for one of the target antigens and (iii) through ADCC mechanisms that interact additively with IT action. We have previously proposed that combination IT therapy would be one means of overcoming the problem of heterogeneity of antigen expression within a global tumour cell population and these additional findings support this and provide a further strengthening of the rationale for employing cocktails of ITs for the treatment of human malignancies. © 2001 Cancer Research Campaign http://www.bjcancer.com


British Journal of Cancer (2001) **84**(4), 571–578
© 2001 Cancer Research Campaign
doi: 10.1054/ bjoc.2000.1633, available online at http://www.idealibrary.com on IDEAL®

### Therapy of human T-cell acute lymphoblastic leukaemia with a combination of anti-CD7 and anti-CD38-SAPORIN immunotoxins is significantly better than therapy with each individual immunotoxin

**DJ Flavell, DA Boehm, A Noss, SL Warnes and SU Flavell**

The Simon Flavell Leukaemia Research Unit, Division of Cancer Sciences, University of Southampton Medical School, Southampton General Hospital, Tremona Rd., Southampton, Hampshire, SO16 6YD, UK

**Summary** Severe combined immunodeficient (SCID) mice injected i.v. with the human T-ALL cell line CCRF CEM (SCID-CEM mice) develop within 50 days life-threatening multi-organ growth of leukaemia cells. The development of leukaemia in SCID-CEM mice treated with three 10 μg i.v. doses of the anti-CD7 immunotoxin (IT) HB2-SAPORIN or the anti-CD38 IT OKT10-SAPORIN was significantly delayed compared with PBS sham-treated animals but 90% of animals treated with either IT eventually developed disseminated leukaemia cell growth. In contrast treatment of SCID-CEM mice with a combination of both ITs led not only to a significantly greater delay in time to leukaemia development but also in the numbers of animals remaining leukaemia free (60%). The native HB2 and OKT10 antibodies (both murine $IgG_1$ antibodies) exerted significant, though relatively weak therapeutic effects, probably mediated through an antibody-dependent cellular cytotoxicity (ADCC) mechanism. Moreover, there was no in vivo additivity of therapeutic effect when both antibodies were used in combination. Apparent, however, was that the combination of HB2-SAPORIN IT with OKT10 antibody led to an intermediate therapeutic effect that was significantly greater than that obtained when either was used alone but significantly less than that obtained when the two IT combination was utilized. This was similarly the case for the combination of OKT10-SAPORIN IT with HB2 antibody though the effect was less pronounced in this instance. This result suggests that the therapeutic effect of IT + antibody treatment results from an additivity between antibody-mediated delivery of saporin combined with a SCID mouse NK cell-mediated ADCC attack on the target cell directed through target cell bound antibody Fc engagement with FcγRIII on the NK cell surface. The combination of both ITs however gave the best therapeutic outcome in SCID-CEM mice probably as the result of (i) delivery of greater amounts of saporin to target CEM cells positive for both CD7 and CD38, (ii) delivery of an effective dose of saporin to CEM cells downregulated or negative for one of the target antigens and (iii) through ADCC mechanisms that interact additively with IT action. We have previously proposed that combination IT therapy would be one means of overcoming the problem of heterogeneity of antigen expression within a global tumour cell population and these additional findings support this and provide a further strengthening of the rationale for employing cocktails of ITs for the treatment of human malignancies. © 2001 Cancer Research Campaign htt://www.bjcancer.com

**Keywords**: T-cell leukaemia; combination immunotoxin therapy; SCID mouse

Immunotoxins (IT) are hybrid molecules comprised of an antibody coupled chemically or genetically to a toxin or ribosome inactivating protein (RIP) such as saporin (Flavell, 1998; Kreitman, 1999; Frankel et al, 2000). The antibody component allows for the selective delivery of the toxin to the surface of cells bearing the target antigen. In order to kill the target cell it is an absolute requirement that the conjugate is internalized by receptor-mediated endocytosis and that subsequently the toxin component is routed to the appropriate intracellular compartment where translocation of the toxin component to the cytosol can take place. Once in the cytosol RIPs such as saporin catalytically inactivate 28S ribosomal RNA via a highly specific N-glycosidase enzymatic activity (Endo, 1988) that results in cessation of protein synthesis in the cell and leads to apoptotic cell death (Bergamaschi et al, 1996).

*Received 15 March 2000*
*Revised 26 October 2000*
*Accepted 28 October 2000*

*Correspondence to:* D Flavell

Arguably, the most important factor that theoretically limits the therapeutic efficacy of immunotoxins that have no bystander effect and which therefore have to exert their cytotoxic influence directly on all cells in the tumour, is the heterogeneity of target antigen expression within the global tumour cell population. Thus, relatively small numbers of tumour cells down-regulated or negative for the target antigen would escape killing by any single immunotoxin and if these surviving cells possess growth potential this would lead to subsequent tumour re-growth. One way of overcoming this problem would be to simultaneously target against more than one target molecule on the tumour cell surface in the expectation that multiple antigen-negative tumour cells would occur with a much lower frequency than single antigen-negative cells. There would also be the added bonus that targeting against more than one antigen on the tumour cell surface would ensure delivery of greater and therefore more effective quantities of the cytotoxic agent carried by antibody to tumour cells that were multiple antigen positive. Other factors such as the cell signalling effects and recruitment of cytotoxic host effectors may also work in conjunction to achieve an improved therapeutic effect when combinations of antibody-based therapeutics are utilized. We have previously shown that therapy outcome in SCID mice xenografted with the human B-cell lymphoma cell line Ramos is significantly improved when a combination of anti-CD19 and anti-CD38 saporin ITs are used compared with when either IT is used individually (Flavell et al, 1995). Indeed we have further shown that a 3 IT combination with anti-CD19, -CD22 and -CD38 specificities can completely cure SCID-Ramos mice (Flavell et al, 1997). Studies by others have similarly shown that combinations of ITs are more effective (Lambert et al, 1985; Strong et al, 1985). These findings allow us to feel optimistic that such a therapeutic approach may be of real practical benefit in achieving better and more robust response rates in patients with this type of immunotherapeutic and justify further preclinical evaluation. In the present study we demonstrate that a combination of anti-CD7 and anti-CD38-saporin ITs are significantly more effective therapeutically in SCID mice xenografted with the human CD7+ CD38+ T-ALL cell line CCRF CEM than the individual ITs when used individually, thus confirming in a completely different tumour system our original findings with the B-cell lymphoma cell line Ramos (Flavell et al, 1995, 1997) and therefore further reinforcing the rationale for developing this approach for clinical trials.

### MATERIALS AND METHODS

### SCID mice

Pathogen free CB.17 scid/scid (SCID) mice (Bosma et al, 1983) of both sexes 6–10 weeks of age were produced from our own breeding colony and used in all the experimental work described here. All animals described in these studies are strictly maintained according to British Home Office regulations under the Animals (Scientific Procedures) Act 1986. The breeding colony is maintained under sterile conditions inside a laminar flow isolator and animals are housed on sterile bedding and provided with sterile water and food ad libitum. Animals for experimental use were transferred out from the isolator to autoclaved filter top micro isolator cages and housed on sterile bedding as 5 single sex animals per cage. These animals were also provided with sterile water and food ad libitum and all manipulations with these animals were carried out in a laminar flow hood by personnel using aseptic techniques. During all procedures involving animals the UKCCCG Guidelines for the Welfare of animals in Experimental Neoplasia were strictly adhered to minimize stress or suffering (Workman et al, 1998).

### CCRF CEM human T-cell acute lymphoblastic leukaemia cell line

The cell line CCRF CEM cell line was derived from the peripheral blood of a paediatric patient with T-cell acute lymphoblastic leukaemia (Foley et al, 1965) and obtained from the American Tissue Culture Collection (ATCC, Bethesda, MD). Cells were maintained in the logarithmic phase of growth in antibiotic-free RPMI 1640 medium containing 10% fetal calf serum and supplemented with 1 mM sodium pyruvate and glutamine (hereafter referred to as R10 medium).

### Antibody production

The hybridoma clone HB2 producing anti-CD7 antibody was produced by culturing HB2 hybridoma cells in an Endotronics Acusyst R hollow fibre bioreactor system (Endotronics Inc, Minneapolis, MN). The anti-CD38 antibody OKT10 was produced by culturing OKT10 hybridoma cells on an Integra Technomouse hollow fibre system (Integra Biosciences, Cramlington, UK) as per manufacturer's instructions. Both antibodies are of the $IgG_1$ subclass. Antibody was purified from culture supernatants by DEAE ion exchange chromatography and size exclusion chromatography on Sepharose S200HR. $F(ab)_2$ fragments of each antibody were produced by pepsin digestion utilizing a kit from Pierce (Perbio Sciences UK Ltd, Tattershall). The specificity of each antibody or respective $F(ab)_2$ fragment was confirmed by flow cytometry and by immunohistochemical staining of sections of frozen tonsil.

### Saporin production

Seeds of the Soapwort plant *Saponaria officinalis* were kindly supplied by Chiltern Seeds, Ulverston, Cumbria, UK. Saporin was extracted from seeds by the method of Stirpe (Stirpe et al, 1983) and purified to homogeneity by a combination of cation exchange chromatography on carboxymethyl-Sepharose and gel filtration on Sephacryl-S200HR (Sigma Chemical Co, Poole, UK). The final product gave a single band of 29 500 daltons on SDS-PAGE and was immunoreactive on ELISA with both polyclonal and monoclonal anti-saporin antisera.

### Immunotoxin construction

The immunotoxins HB2-SAPORIN (anti-CD7) and OKT10-SAPORIN (anti-CD38) were constructed by conjugating HB2 or OKT10 antibody to saporin with the heterobifunctional cross linking reagent N-succinimidyl 3-(2-pyridyldithio) propionate (SPDP) (Pharmacia, Uppsala, Sweden) as described previously (Thorpe et al, 1985). Immunotoxins prepared in this way contained a non-hindered disulphide bond between antibody and saporin. Free antibody was removed from the immunoconjugates by carboxymethyl-Sepharose (Sigma Chemical Co, Poole, UK) cation exchange chromatography as described previously (Lambert et al, 1985). Purified immunotoxins were dialysed into PBS pH 7.2, sterilized by passage through a 0.2 μm filter and stored deep frozen in 100 μg aliquots at −80˚C.

### Flow cytometry

Dual colour analysis for the cell surface expression of CD7 and CD38 by CCRF CEM cells was measured on an EPICS XL flow cytometer (Beckman Coulter UK, High Wycombe) equipped with Coulter PC-based analytical software. CCRF cells were first stained at 4˚C with OKT10 antibody (10 μg $ml^{-1}$ in PBS containing 0.1% sodium azide) followed by a 1:20 dilution of FITC labelled $F(ab)_2$ goat anti-mouse immunoglobulin (Sigma Chemical Co, Poole, Dorset). Cells were then double-labelled by incubation for a further 30 minutes at 4˚C with biotinylated HB2 antibody (10 μg $ml^{-1}$) in PBS also containing 0.1% sodium azide. Cells were then washed twice and phycoerythrin-labelled streptavidin (Dako, High Wycombe, Bucks) at a 1:100 dilution was incubated with the cell pellet for 20 min at 4˚C after which analysis was carried out on a Coulter EPICS XL flow cytometer.

Single colour flow cytometric analysis was similarly conduced on single cell suspensions of solid CCRF CEM tumours removed from IT or PBS sham-treated animals but using HB2 and OKT10 antibodies directly labelled with FITC.

 © 2001 Cancer Research Campaign

### SDS-PAGE analysis

SDS PAGE analysis (Laemmli, 1970) was used to confirm the purity of antibody, saporin and ITs. 5% non-reducing gels with 3% stacks were routinely used for separations.

### Protein synthesis inhibition assay

The ability of each IT, native antibody or saporin to inhibit protein synthesis in target CCRF CEM cells was evaluated by a [$^3$H]-leucine incorporation assay as described previously (Flavell et al, 1995) (CN Pharmaceuticals Ltd, Basingstoke).

### Cell outgrowth assay

CCRF CEM cells ($1 \times 10^5$ cells) were cultured in R10 medium in T25 culture flasks in the presence of given concentrations of IT, antibody or combinations of both. Control cultures were grown in R10 medium only. Viable haemocytometer cell counts using Trypan blue exclusion were carried out on cultures at regular intervals.

### Chromium release assay

$^{51}$Cr (Amersham-Pharmacia, Amersham) release assays were performed as described previously (Flavell et al, 1998) in order to determine and quantify the extent of complement-dependent cytotoxicity (CDC) or antibody-dependent cellular cytotoxicity mediated by each antibody or each IT construct against target Ramos cells.

### Establishment of CCRF CEM leukaemia in SCID mice

Two million CCRF CEM cells were injected into the tail vein (i.v.) of SCID mice in a 200 μl volume of R10 medium. In all therapy studies cells were injected 7 days prior to commencing treatment with IT or antibody.

### Therapy protocols

7 days after i.v. injection of CCRF CEM cells, appropriate groups were treated with three 10 μg doses of either IT, antibody or a combination of both given as a bolus injection (in a 200 μl volume of PBS) into a tail vein on alternate days (i.e. days 7, 9 and 11). Where a combination of two ITs, two antibodies or an IT plus an antibody was used the 10 μg dose was comprised of 5 μg of each component part. For antibody treatment the dose was comprised of 8 μg antibody plus 2 μg saporin, this ratio being approximately representative of the molar equivalent content of each component contained within the 10 μg dose of IT.

All animals were monitored twice daily for the duration of the study and strictly defined endpoints were enforced as per recommendations under the UKCCCR guidelines (Workman et al, 1998) to ensure that no animal suffered undue or unnecessary stress or pain during the course of the study. Thus, animals showing any early identifiable signs of becoming unwell were killed humanely and full post-mortem examinations conducted to confirm the presence of tumour.

### Statistical analysis

Log-Rank analysis (Peto's method) using the SOLO statistics software package (BMDP Statistical Software, Los Angeles, CA, USA) was used to determine if there were any significant differences between the various therapy groups. *P* values of 0.05 or less were considered as statistically significant in these studies.

### Histopathology

All tissues obtained at post-mortem from animals sacrificed intercurrently or killed electively at termination of the experiment were fixed in 10% neutral buffered saline (NBFS), processed and embedded in paraffin wax. Sections were cut and stained with haematoxylin and eosin (H&E) or immunostained with the anti-CD43 antibody DF-T1 that identifies human T-cells in paraffin section (Stross et al, 1989).

### RESULTS

### Growth of CCRF CEM leukaemia in SCID mice

The pattern of growth of CCRF CEM in non-conditioned SCID mice was virtually identical to that described by us previously for the growth of HSB-2, another human T-ALL cell line (Morland et al, 1994). In summary, SCID mice injected with two million CCRF CEM cells developed multi-organ involvement with observable growth of solid CCRF CEM tumours in liver, kidney, bone marrow, meninges and elsewhere. Animals injected with this number of CCRF CEM cells were electively killed between 36 and 49 days when early symptoms of disease appeared. The presence of CCRF CEM tumour growth was confirmed in these animals by post mortem and where necessary (i.e. when no gross tumour was visible) by histopathology and immunohistochemical staining with the anti-CD43 antibody DF-T1. SCID-CEM mice from the different treatment regimes that were killed inter-currently during the course of the experiment all had demonstrable tumour growth. Animals that survived to 300 days that were electively killed at this time showed no gross or microscopic evidence of tumour growth.

### Expression of CD7 and CD38 by CCRF CEM cells

The results of two-colour flow cytometric analysis of CCRF CEM cells for CD7 and CD38 expression are shown in Figure 1. The vast majority (99.59%) of CEM cells possessed a double antigen positive (CD7+ CD38+) immunophenotype with 0.04% with a CD7+ CD38- and 0.29% with a CD7-CD38+ immunophenotype. A tiny subpopulation of 0.09% were double antigen negative (CD7- CD38-).

Single cell suspensions of CCRF CEM tumours growing in two SCID-CEM mice from each group treated with each individual IT with a combination of both ITs or sham-treated with PBS were stained with HB2 or OKT10 antibody for flow cytometry. CCRF CEM tumours from the IT-treated animals were positive for CD7 and CD38 expression at similar levels exhibited by cells taken from PBS sham-treated control animals (data not shown) and we can conclude therefore that either single or combination IT treatment did not select for antigen-negative tumour cell variants.

### Lysis of Ramos cells by HB2 and OKT10 antibody and respective IT constructs in CDC and ADCC

Chromium release assays were used to determine whether HB2 and OKT10 antibody or their respective IT constructs were

© 2001 Cancer Research Campaign

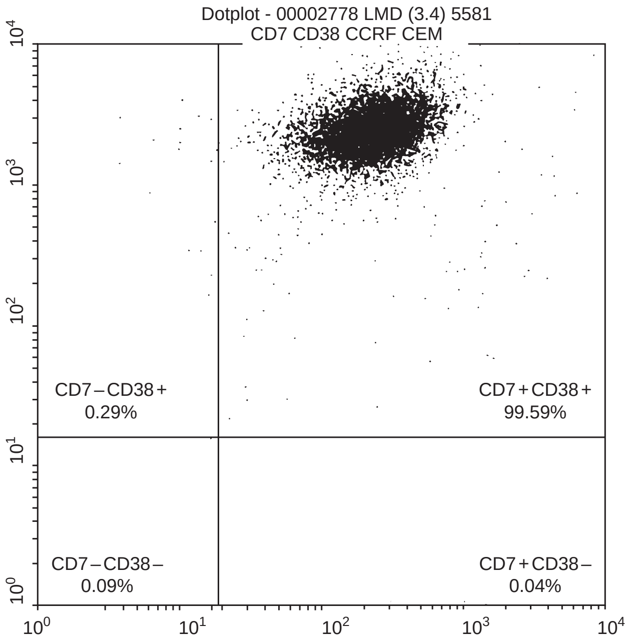

**Figure 1** Two colour flow cytometric analysis of CD7 and CD38 expression by the human T-cell acute lymphoblastic leukaemia cell line CCRF CEM. The percentage of cells with a given immunophenotypic expression of CD7/CD38 is shown in each quadrant

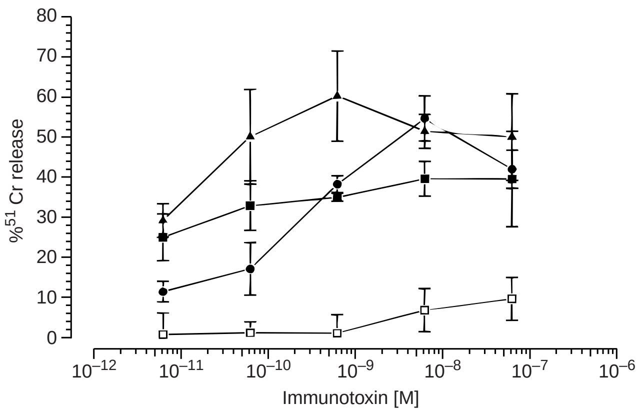

**Figure 2** Lysis of CCRF CEM target cells in an ADCC chromium release assay utilizing SCID mouse splenocytes as effectors (target:effector ratio 1:70) in the presence of increasing molar concentrations of HB2-SAPORIN IT (■), OKT10-SAPORIN IT (●), a combination of both ITs (▲), or the irrelevant anti-CD19 IT BU12-SAPORIN (□)

capable of eliciting complement-dependent cytotoxicity (CDC) via the classical pathway following antibody binding to the target cell surface or antibody-dependent cellular cytotoxicity (ADCC) mediated by SCID mouse splenocytes as effectors. Antibodies or ITs used singly or in pair combinations had no lytic activity in the CDC assay system. In contrast very clear antibody or IT dose-dependent lysis of CCRF CEM cells occurred when SCID mouse splenocytes were included in an ADCC assay (target cell:effector cell ratio of 1:70) (Figure 2). HB2-SAPORIN and OKT10-SAPORIN ITs each exerted similar lytic activities in ADCC though there was a greater dose dependency seen with OKT10-SAPORIN (Figure 2). Both ITs used in combination exerted greater lytic activity than each individual IT particularly at the mid-level of the concentration range studied. An irrelevant IT, BU12-SAPORIN identifying the CD19 molecule that is not expressed by CRF CEM cells did not cause any lysis of these cells in ADCC. Near identical lysis profiles were obtained when intact HB2 and OKT10 antibodies were used in place of the respective IT (data not shown). A $F(ab)_2$ fragment of each antibody used over the same molar concentration range did not cause lysis of CCRF CEM cells in ADCC demonstrating an absolute requirement for the Fc domain of antibody for lysis (data not shown).

### Inhibition of protein synthesis in CCRF CEM cells by immunotoxins and antibodies

The effects of increasing molar concentrations of HB2-SAPORIN IT, OKT10-SAPORIN IT, HB2 antibody and OKT10 antibody used individually or in various combinations on protein synthesis levels in target CCRF CEM cells are summarized in Table 2. OKT10-SAPORIN performed best against CCRF CEM cells with an achieved $IC_{50}$ of $3.8 \times 10^{-11}$ M representing a 2895-fold increase over saporin. HB2-SAPORIN gave a 1549-fold increase with an achieved $IC_{50}$ of $7.1 \times 10^{-11}$ M. A mixture of both immunotoxins performed in an intermediate fashion with an achieved $IC_{50}$ of $5.9 \times 10^{-11}$ M (1864-fold increase). HB2 or OKT10 antibodies used individually or in combination over the same concentration range had no significant effect on protein synthesis levels in CCRF CEM cells (data not shown). When a mixture of OKT10-SAPORIN plus HB2 antibody or HB2-SAPORIN plus OKT10 antibody was employed, performance was

**Table 1** Summary of experimental SCID mouse groups and therapy outcomes

| Treatment group | Treatment | No. animals | Mean survival (days)[a] | % Survivors[b] |
|---|---|---|---|---|
| a | PBS | 10 | 40.5 | 0 |
| b | HB2-SAPORIN IT | 10 | 83.7 | 10 |
| c | OKT10-SAPORIN IT | 10 | 109.0 | 10 |
| d | HB2-SAPORIN + OKT10-SAPORIN ITs | 10 | 231.6 | 60 |
| e | HB2 Ab | 10 | 47.0 | 0 |
| f | OKT10 Ab | 10 | 94.6 | 10 |
| g | HB2 Ab + OKT10 Ab | 10 | 90.6 | 10 |
| h | HB2-SAPORIN IT + OKT10 Ab | 10 | 163.6 | 40 |
| i | OKT10-SAPORIN IT + HB2 Ab | 10 | 137.5 | 20 |

[a]Mean survival calculated at termination of study at 300 days. [b]Percentage survivors at 300 days.

 © 2001 Cancer Research Campaign reduced to a level below that seen when each IT was used individually, with achieved $IC_{50}$ values of $8.8 \times 10^{-11}$ M and $1.5 \times 10^{-10}$ M, respectively (Table 2).

### Inhibition of CCRF CEM cell growth by immunotoxins and antibodies

The results for long-term outgrowth of CCRF CEM cells exposed continuously to HB2-SAPORIN or OKT10-SAPORIN at a concentration of 1 μg $ml^{-1}$ or to a combination of both ITs each at 0.5 μg $ml^{-1}$ (i.e. 1 μg $ml^{-1}$ total) are shown in Figure 3. Each individual IT used at this concentration delayed the growth of CCRF CEM cells by between 20 to 30 days compared to medium controls but growth did eventually occur by this time. In contrast, growth of CCRF CEM cells was completely inhibited when cells were exposed to an equivalent total concentration of a combination of both ITs with no living cells detectable in these cultures at day 50. CCRF CEM cells growing out following exposure to either individual IT still expressed CD7 and CD38 at near normal levels and were still sensitive to the cytotoxic effects of both ITs in protein synthesis inhibition assays (data not shown). HB2 or OKT10 antibody used individually or in combination at the same total concentration of 1 μg $ml^{-1}$ had no significant effect on CCRF CEM growth (data not shown). The combination of HB2-SAPORIN with OKT10 antibody or OKT10-SAPORIN with HB2 antibody were both less effective at inhibiting CCRF CEM outgrowth than were the individual ITs with cell growth occurring earlier (Figure 3).

### Immunotoxin and antibody therapy of SCID mice with disseminated CCRF CEM leukaemia

The therapeutic effects of the anti-CD7 IT, HB2-SAPORIN or of the anti-CD38 IT, OKT10-SAPORIN or of native antibody used individually or in combination, or a combination of a single immunotoxin with a single antibody were investigated in groups of SCID mice bearing disseminated CCRF CEM leukaemia. Details of the various therapy groups are given in Table 1 together with a summary of the mean survival times and percentage survivors in each treatment group. A comparison made between the different therapy groups for statistically significant differences was undertaken by log-rank analysis and these data are shown in Table 3.

The Kaplan-Meier curves obtained for SCID-CEM mice treated with each individual IT or a combination of both ITs are shown in Figure 4. Control animals (group a) given PBS sham-treatment all developed symptomatic disseminated CCRF CEM leukaemia by 49 days. Treatment with HB2-SAPORIN (group b) led to a significant prolongation in time to symptomatic disease appearance (mean time of 83.7 days) compared with controls ($P = 0.0012$) though 90% of these animals had eventually developed symptomatic disseminated leukaemia by day 81. OKT10-SAPORIN (group c) gave a more prolonged survival time (mean 109 days) compared with the PBS sham-treated control group. When both ITs were used in combination (group d) there was a highly significant improvement in both mean survival time (231.6 days) and in the proportion of animals that remained disease-free (60% at 300 days). The difference between the PBS sham-treated group (group a) and the combination IT group (group d) was highly significant ($P = 0.0003$).

We also examined the therapeutic effects of the native HB2 and OKT10 antibodies in the SCID-CEM model (Tables 1 and 3). HB2 antibody exerted a small, though significant ($P = 0.0293$), therapeutic effect in SCID-CEM mice (group e) prolonging the time to

**Table 2** In vitro inhibition of protein synthesis in target CEM cells by immunotoxins used singly or in combination or by a single immunotoxin used in combination with a single antibody

| Treatment | $IC_{50}$ (M)[a] | Fold-increase[b] |
|---|---|---|
| HB2-SAP IT | $7.1 \times 10^{-11}$ | 1549 |
| OKT10-SAP IT | $3.8 \times 10^{-11}$ | 2895 |
| HB2-SAP IT + OKT10-SAP IT | $5.9 \times 10^{-11}$ | 1864 |
| HB2-SAP IT + OKT10 Ab | $1.5 \times 10^{-10}$ | 733 |
| OKT10-SAP IT + HB2 Ab | $8.8 \times 10^{-11}$ | 1250 |
| SAPORIN ALONE | $1.1 \times 10^{-7}$ | – |

[a]$IC_{50}$ calculated as the molar concentration of IT needed to achieve 50% inhibition of $^3$H-leucine incorporation compared with untreated control cells. [b]Fold increase calculated as $IC_{50}$ saporin alone/$IC_{50}$ treatment.

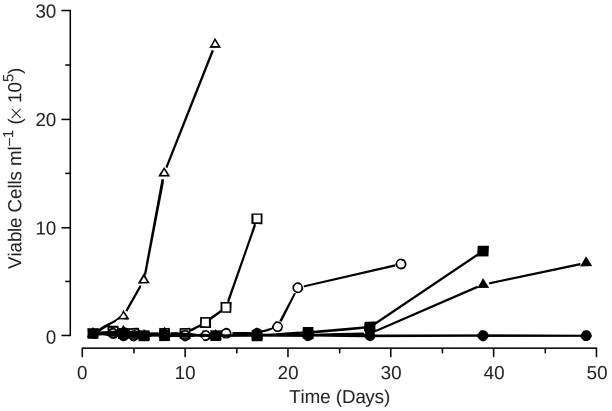

**Figure 3** Outgrowth of CCRF CEM cells in the continuous presence of HB2-SAPORIN (■), OKT10-SAPORIN (▲), a combination of both HB2-SAPORIN + OKT10-SAPORIN (●) a combination of HB2-SAPORIN + OKT10 antibody (□), OKT10-SAPORIN + HB2 antibody (○) or medium control (△). Cultures exposed to a single reagent at a concentration of 1 μg $ml^{-1}$. When a combination of two reagents was employed the final concentration of both reagents combined was 1 μg $ml^{-1}$ (i.e. 0.5 μg $ml^{-1}$ of each)

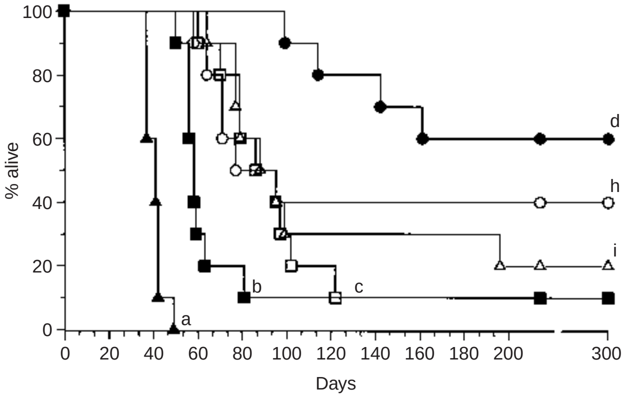

**Figure 4** Kaplan-Meier plots for SCID-CEM mice injected on day 1 with two million CCRF CEM cells and treated with 3 i.v. injections of 10 μg each (given on days 7, 9 and 11) of HB2-SAPORIN (■), OKT10-SAPORIN (□), a combination of both ITs (●) HB2-SAPORIN + OKT10 antibody (○), OKT10-SAPORIN + HB2 antibody (△), or PBS sham-treated (▲). Where animals received a combination of two reagents totalling 10 μg dose this was comprised of 5 μg of each component. Group identification letters, refer to Table 1

© 2001 Cancer Research Campaign

**Table 3** Comparison of the various treatment groups for mean survival and for statistically significant differences by Log-Rank analysis

| Treatment 1 | vs | Treatment 2 | Mean survival (days) | | | *P* value |
|---|---|---|---|---|---|---|
| | | | 1 | vs | 2 | |
| a | | b | 40.5 | | 83.7 | 0.0012 |
| a | | c | 40.5 | | 109.0 | 0.0015 |
| a | | d | 40.5 | | 231.6 | 0.0003 |
| a | | e | 40.5 | | 47.0 | 0.0293 |
| a | | f | 40.5 | | 94.6 | 0.0018 |
| a | | g | 40.5 | | 90.6 | 0.0022 |
| b | | c | 83.7 | | 109.0 | 0.0697 |
| b | | d | 83.7 | | 231.6 | 0.0028 |
| c | | d | 109.0 | | 231.6 | 0.0043 |
| b | | e | 83.7 | | 47.0 | 0.0180 |
| c | | f | 109.0 | | 94.6 | 0.0015 |
| d | | g | 231.6 | | 90.6 | 0.0033 |
| e | | g | 47.0 | | 90.6 | 0.0203 |
| e | | f | 47.0 | | 94.6 | 0.0195 |
| g | | f | 90.6 | | 94.6 | 0.9990 |
| d | | h | 231.6 | | 163.6 | 0.1698 |
| d | | I | 231.6 | | 137.5 | 0.0286 |
| h | | I | 163.6 | | 137.5 | 0.7656 |
| i | | g | 137.5 | | 90.6 | 0.1478 |

symptomatic leukaemia appearance to 47 days but with all animals developing leukaemia by day 78. Comparison of HB2 antibody-treated animals with HB2-SAPORIN treated animals showed that the therapeutic effect of this IT was significantly greater than the antibody-mediated effect ($P = 0.018$). OKT10 antibody (group f) exerted a better and more significant therapeutic effect than HB2 antibody, prolonging the mean time to symptomatic leukaemia appearance for animals in this group to 94.6 days with a significance level of $P = 0.0015$ when compared with PBS sham-treated control animals. The therapeutic effect of OKT10 antibody was however significantly less than that exerted by the OKT10-SAPORIN IT ($P = 0.0015$). The time to leukaemia appearance characteristics of animals treated with a combination of HB2 and OKT10 antibodies (group g) was virtually identical to that for those animals treated with OKT10 antibody alone and there was therefore no therapeutic gain to be had from treating with a combination of both antibodies.

In order to ascertain whether it might be an additive interaction between one of the IT components and one of the antibody components present in the two combination IT treatment that led to the significant improvement in therapeutic effectiveness, we undertook studies in which groups of SCID-CEM mice were treated with an IT/antibody pair. The results of this study are also shown in Figure 4. The therapeutic effectiveness of HB2-SAPORIN + OKT10 antibody (group h) or OKT10-SAPORIN + HB2 antibody (group i) treatments was significantly below that obtained for the combination of both ITs C (group d). However, the combination of HB2-SAPORIN IT. OKT10 antibody (group h) did significantly increase the therapeutic effects above that obtained when either HB2-SAPORIN IT (group b) or OKT10 antibody (group f) were used alone.

### DISCUSSION

The main finding to emerge from this study is that two saporin immunotoxins directed against the CD7 and CD38 antigens on the surface of the human T-cell acute lymphoblastic leukaemia cell line CCRF CEM, perform significantly better therapeutically in SCID-CEM mice when used in combination than when used individually. Those leukaemias that did eventually emerge in the two IT cocktail still expressed both target antigens demonstrating that therapy had not selected for antigen-negative variants as has been the case with anti-idiotypic antibodies directed against the cell surface immunoglobulin of B-cell tumours (Glennie et al, 1987). As we have argued previously antigen-expressing tumour cells may have avoided killing by IT either because a small subpopulation of the global tumour cell population were epigenetically down-regulated for antigen expression at the time of treatment or alternatively because small numbers of leukaemia cells were at locations inaccessible to IT at the time of treatment. We reason that it was from these ‘escapee’ cells that tumours eventually emerged. The in vivo findings were also reflected in a long term in vitro outgrowth assay where the combination of two ITs completely inhibited target cell proliferation. However, this was not the case in a short-term protein synthesis inhibition assay where there was actually a reduction in apparent cytotoxicity toward CEM cells when a combination of two ITs was employed. Curiously, as exemplified in the present and other studies (Flavell et al, 1997), we have never been able to show any improvement in $IC_{50}$ values when combinations of ITs are used against target cells expressing the appropriate target antigens.

The present study has confirmed in a different model tumour system very similar findings, which we originally made with the human B-cell lymphoma cell line Ramos when targeted against with a combination of anti-CD19 and -CD38-saporin ITs (Flavell et al, 1995). In the instance of the SCID-Ramos model it was found that the improved therapeutic benefit was entirely due to an immunotoxin-mediated effect, as combination pairs of IT + antibody (i.e. anti-CD19 IT + anti-CD38 antibody or anti-CD38 IT + CD19 antibody) did not improve the therapeutic outcome. This finding was contrary to that of Ghetie et al (1992) who were able to demonstrate that the anti-CD19 antibody HD37 used in combination with an anti-CD22 ricin A chain IT (RFB4-dgR) was equally as effective therapeutically in SCID mice xenografted with the human B-cell lymphoma cell line Daudi as was a specificity equivalent combination of two ITs. Additional work from the same

*© 2001 Cancer Research Campaign*

group showed that it was the anti-proliferative signalling effects of CD19 antibodies in conjunction with toxin-mediated cytotoxicity delivered via an anti-CD22 antibody that actually augmented the therapeutic efficacy (Ghetie et al, 1994).

In separate, though related studies we have also shown that the anti-CD7 immunotoxin HB2-SAPORIN exerts its therapeutic effects against the human T-ALL cell line HSB-2 growing in SCID mice via two separate mechanisms; firstly through delivery of the rip saporin to cells expressing the target CD7 molecule on their surface and secondly through an antibody-dependent cellular cytotoxicity (ADCC) mechanism mediated by SCID mouse NK or NK-like cells (Flavell et al, 1998). It is also evident in the present study that HB2 (anti-CD7) antibody exerted a small therapeutic effect in SCID-CEM mice manifest as a significant increase in the time to symptomatic leukaemia appearance but without being capable of effecting cures. OKT10 antibody performed somewhat better achieving a significantly greater prolongation in time to symptomatic leukaemia appearance but with 90% of the animals still eventually developing disseminated leukaemia. Both of these naked antibodies probably mediate their in vivo therapeutic effects via ADCC and the in vitro chromium release results presented here show demonstrably that both antibodies and ITs can evoke ADCC. These results are very similar to those obtained with the anti-CD19 antibody BU12 and same anti-CD38 antibody in SCID mice bearing the CD19+ CD38+ human B-cell lymphoma cell line Ramos (Flavell et al, 1995). In both described tumour systems it therefore appears that anti-CD38 antibody performs therapeutically better than either the anti-CD19 antibody in the SCID-Ramos model, or the anti-CD7 antibody in the SCID-CEM system. As all 3 antibodies belong to the same subclass ($IgG_1$) and as expression levels of all 3 target molecules are very similar, it is therefore unclear why antibody directed against CD38 on the tumour cell surface should be more effective than anti-CD7 antibody. It may be that the topography of CD38 on the cell surface or its manner of insertion into the membrane renders the target cell more susceptible to cytolysis by ADCC directed against this molecule. In a previous study in SCID-Ramos mice we demonstrated that a combination pair of anti-CD19 IT + anti-CD38 antibody or anti-CD38 IT plus anti-CD19 antibody did not exert any improved therapeutic effect above that seen with the individual IT present in the cocktail (Flavell et al, 1995). Thus, we concluded that the significantly improved therapeutic effect obtained with the combination of anti-CD19 and anti-CD38 ITs in SCID-Ramos mice was not due to antibody signalling such as has been described for the SCID-Daudi model (Ghetie et al, 1992) or due to cytotoxic effector recruitment exerted by the antibody component combined with cytotoxicity due to toxin delivery via IT (Flavell et al, 1998).

The current results described in this SCID-CEM model are therefore somewhat different than our previous findings in the SCID-Ramos model. In the current study it is clear that a combination of anti-CD7 IT plus anti-CD38 antibody improves the therapeutic outcome, though the effect is still significantly lower than that obtained when the two specificity equivalent ITs are used in combination. A similar effect was observed with anti-CD38 IT combined with anti-CD7 antibody but this was less pronounced. Having previously shown that it is ADCC mediated by SCID mouse NK or NK-like cells that acts additively with the anti-CD7 IT HB2-SAPORIN IT to achieve the final therapeutic effect in SCID mice bearing the human T-cell leukaemia HSB-2 (Flavell et al, 1998) it is entirely possible that a similar effect is operating in the described SCID-CEM model. Indeed the in vitro results presented here demonstrate that both naked antibody and equivalent ITs can efficiently mediate the lysis of CCRF CEM cells in ADCC but not CDC obviating a role for complement. Neither is there any direct evidence from the data presented here that direct cell signalling mediated by ligation of CD7 or CD38 by antibody has any significant influence on CCRF CEM cell proliferation or viability. We therefore hypothesize that it must be a host effector mechanism(s) such as ADCC driven by engagement of SCID mouse NK cell FcγRIII with the Fc portion of the antibody component of the HB2-SAPORIN or OKT10-SAPORIN IT in conjunction with saporin-mediated cytotoxicity delivered by IT that is likely responsible for the improved therapeutic performance of the IT + antibody combinations. This hypothesis is further strengthened by the observation made in the present study that lysis of CCRF CEM cells is increased when combination pairs of ITs or antibodies are employed. However, the fact that the therapeutic effect of the IT + antibody combination is significantly less than that obtained with the two IT combination indicates that other factors must be contributing to the therapeutic mechanism operative with the two IT combination. In conclusion we hypothesize that the combination of the two ITs used in the current study recognizing CD7 or CD38 exert an improved therapeutic efficacy for any one or possibly all of the following individual reasons: (i) that greater and therefore presumably more effective quantities of saporin are delivered to CEM cells that express *both* the CD7 and CD38 target molecules, (ii) that an effective dose of saporin is still delivered to those CEM cells that are down-regulated or negative for a single target antigen and (iii) that ADCC mechanisms may exert their influence more effectively when two different cell surface molecules are targeted against and that this may then work additively together with the selective target cell cytotoxicity delivered by IT. It must be kept in mind that this is a model system that has provided some proof of principle but that ITs constructed with mouse $IgG_1$ would not necessarily efficiently recruit human effector cells (ie human FcγRIII bearing NK-cells and macrophages). Therefore some of the beneficial effects described here with mouse antibody-based molecules would not operate in human disease. To achieve this it would be necessary to engineer into the IT constructs human Fc domains that could efficiently recruit human effectors.

We have previously proposed that combination IT therapy would be one means of overcoming the problem of heterogeneity of antigen expression within a global tumour cell population. These additional findings support this hypothesis and provide a further strengthening of the rationale for employing cocktails of ITs for the treatment of human malignancies.

### ACKNOWLEDGEMENTS

This work was supported by the children's leukaemia research charity Leukaemia Busters. We would like to thank the staff of the Biomedical Research Facility at the University of Southampton for their excellent technical assistance with these studies.

### REFERENCES

Bergamaschi G, Perfetti V, Tonon L, Novella A, Lucotti C, Danova M, Glennie MJ, Merlini G and Cazzola M (1996) Saporin, a ribosome-inactivating protein used to prepare immunotoxins, induces cell death via apoptosis. *Br J Haematol* **93**: 789–794

© 2001 Cancer Research Campaign 

Bosma GC, Custer RP and Bosma MJ (1983) A severe combined immunodeficiency mutation in the mouse. *Nature* **301**: 527–530

Endo Y (1988) The site of action of six different ribosome inactivating proteins from plants on eukaryotic ribosomes: the RNA N-glycosidase activity of the protein. *Biochem Biophys Res Commun* **150**: 1032–1036

Flavell DJ (1998) Saporin immunotoxins. In: *Clinical Applications of Immunotoxins*, Frankel AE (ed), Vol. 234. pp. 57–61. Current Topics in Microbiology and Immunology. Springer: Berlin

Flavell DJ, Boehm DA, Emery L, Noss A, Ramsay A and Flavell SU (1995) Therapy of human B-cell lymphoma bearing SCID mice is more effective with anti-CD19 and anti-CD38-saporin immunotoxins used in combination than with either immunotoxin used alone. *Int J Cancer* **62**: 337–344

Flavell DJ, Noss A, Pulford KAF, Ling N and Flavell SU (1997) Systemic therapy with 3BIT, a triple combination cocktail of anti-CD19, -CD22 and -CD38-saporin immunotoxins is curative of human B-cell lymphoma in SCID mice. *Cancer Res* **57**: 4824–4829

Flavell DJ, Warnes S, Noss A and Flavell SU (1998) Host- mediated antibody dependent cellular cytotoxicity (ADCC) contributes to the *in vivo* therapeutic efficacy of an anti-CD7-Saporin immunotoxin in a SCID mouse model of Human T-ALL. *Cancer Res* **58**: 5787–5794

Foley GE, Lazarus H, Farber S, Uzman BG, Boone BA and McCarthy RE (1965) Continuous culture of human lymphoblasts from peripheral blood of a child with acute leukaemia. *Cancer* **18**: 522–529

Frankel AE, Kreitman RJ and Sausville EA (2000) Targeted toxins. *Clin Cancer Res* **6**: 326–334

Ghetie A.-M, Picker LJ, Richardson JA, Tucker K, Uhr JW and Vitetta ES (1994) Anti-CD19 inhibits the growth of human B cell tumor lines *in vitro* and of Daudi cells in SCID mice by inducing cell cycle arrest. *Blood* **83**: 1329–1336

Ghetie M-A, Tucker K, Richardson J, Uhr JW and Vitetta ES (1992) The antitumour activity of an anti-CD22 immunotoxin in SCID mice with disseminated Daudi lymphoma in enhanced by either an anti-CD19 antibody or an anti-CD19 immunotoxin. *Blood* **80**: 2351–2320

Glennie MJ, McBride HM, Stirpe F, Thorpe PE, Worth AT and Stevenson GT (1987) Emergence of immunoglobulin variants following treatment of a B cell leukaemia with an immunotoxin composed of antidiotypic antibody and Saporin. *J Experimental Medicine* **166**: 43–62

Kreitman RJ (1999) Immunotoxins in cancer therapy. *Curr Opin Immunol* **11**: 570–578

Laemmli UK (1970) Cleavage of structural proteins during the assembly of the head of bacteriophage T4. *Nature* **227**: 680

Lambert JM, Senter PD, Yau-Young A, Blattler WA and Goldmacher VS (1985) Purified immunotoxins that are reactive with human lymphoid cells. *J Biol Chem* **260**: 12035–12041

Morland BJ, Barley J, Boehm D, Flavell SU, Ghaleb N, Kohler JA, Okayama K, Wilkins B and Flavell DJ (1994) Effectiveness of HB2(anti-CD7)-saporin immunotoxin in an *in vivo* model of human T-cell leukaemia developed in severe combined immunodeficient Mice. *Br J Cancer* **69**: 279–285

Stirpe F, Gasperi-Campani G, Barbieri G, Falasca A, Abbondanza A and Stevens WA (1983) Ribosome inactivating proteins from the seeds of *Saponaria officinalis L* (soapwort), of *Agrostemma githago L* (corn cockle) and of *Asparagus officinalis* (asparagus) and from the latex of *Hura crepitans L* (Sandbox tree). *Biochem J* **216**: 617

Strong RC, Uckun F, Youle RJ, Kersey JH and Vallera DA (1985) Use of multiple T-cell-directed intact ricin immunotoxins for autologous bone marrow transplantation. *Blood* **66**: 627–635

Stross WP, Warnke RA, Flavell DJ, Flavell SU, Simmons D, Gatter KC and Mason DY (1989) Molecule detected in formalin fixed tissue by antibodies MT1, DF-T1 and L60 (Leu-22) Corresponds to CD43 Antigen. *J Clin Pathol* **42**: 953–961

Thorpe PE, Brown ANF Jr, JAGB, Foxwell BMJ and Stirpe F (1985) An immunotoxin composed of monoclonal anti-Thy 1.1 antibody and a ribosome-inactivating protein from *Saponaria officinalis*: potent antitumor effects in vitro and in vivo. *J National Cancer Inst* **75**: 151–159

Workman P, Twentyman P, Balkwill F, Balmain A, Chaplin D, Double J, Embleton J, Newell D, Raymond R, Stables J, Stephens T and Wallace J (1998) United Kingdom co-ordinating committee on cancer research (UKCCCR) guidelines for the welfare of animals in experimental neoplasia (second edition). *Br J Cancer* **77**: 1–10

*© 2001 Cancer Research Campaign*
